# Investigation of the involvement of platelet-activating factor in the control of hypertension by aerobic training. A randomized controlled trial

**DOI:** 10.5114/biolsport.2024.131819

**Published:** 2023-11-17

**Authors:** João Paulo Prado, Ana Emilia Castro, Jonatan Carvalho, Daniele Pereira, Lúcia Helena Faccioli, Carlos Sorgi, Rômulo Novaes, Silvia Silva, Giovane Galdino

**Affiliations:** 1 Institute of Motricity of Sciences, Federal University of Alfenas, 2600 Jovino Fernandes Sales Ave, Alfenas, MG 37133-550, Brazil; 2 Department of Chemistry, Faculty of Philosophy, Sciences, and Letters of Ribeirao Preto, Univer-sity of Sao Paulo, Ribeirao Preto, Brazil; 3 Department of Physical Therapy, Universidade Federal de Minas Gerais, Belo Horizonte, Brazil; 4 Faculty of Pharmaceutical Sciences of Ribeirão Preto, Universidade de São Paulo, Ribeirão Preto, Brazil; 5 Department of Structural Biology, Institute of Biomedical Sciences, Federal University of Alfenas, 700 Gabriel Monteiro Silva St, Alfenas, MG 37130-001, Brazil; 6 Faculty of Medicine, Federal University of Juiz de Fora, MG, Brazil

**Keywords:** Exercise, Hypertension, Cytokines, Respiratory muscle strength, Quality of life

## Abstract

Although studies have demonstrated the effectiveness of exercise in controlling systemic arterial hypertension (SAH), the mechanisms involved in this effect are still poorly understood. Thus, this study investigated the impact of aerobic training on the relationship between platelet-activating factor (PAF) circulating levels and blood pressure in hypertensives. Seventy-seven hypertensive subjects were enrolled in this randomized controlled trial (age 66.51 ± 7.53 years, body mass 76.17 ± 14.19 kg). Participants were randomized to two groups: the intervention group (IG, n = 36), composed of hypertensive individuals submitted to an aerobic training protocol, and the control group (CG, n = 41), composed of non-exercised hypertensives. Body mass index, arterial blood pressure, quality of life, respiratory muscle strength, and functional capacity were assessed before and after 12 weeks. PAF and plasma cytokine levels were also evaluated respectively by liquid chromatography coupled with mass spectrometry and enzyme-linked immunosorbent assay. Aerobic training promoted a significant reduction in blood pressure while functional capacity, expiratory muscle strength, and quality of life, PAFC16:0 and PAFC18:1 plasma levels were increased in comparison to the CG (p < 0.05). In addition, multiple correlation analysis indicated a positive correlation [F (3.19) = 6.322; p = 0.001; R^2^adjusted = 0.499] between PAFC16:0 levels and expiratory muscle strength after aerobic training. Taken together, our findings indicate that PAF may be involved in the indirect mechanisms that control SAH, being mainly associated with increased respiratory muscle strength in hypertensive subjects undergoing aerobic training.

## INTRODUCTION

More than 30% of the world population is diagnosed with systemic arterial hypertension (SAH). This disease is the main risk factor for the development of cardiovascular diseases, especially coronary artery disease and stroke [1]. Although controlling SAH is challenging, pharmacotherapy combined with lifestyle changes can reduce the morbidity and mortality associated with this chronic disease [1]. Considering the pharmacological strategies currently available, diuretics, angiotensin-converting enzyme (ACE) inhibitors, angiotensin receptor blockers (ARBs), beta-blockers, and calcium channel blockers (CCBs) are the main drugs used to treat SAH. Furthermore, some patients require two or more antihypertensive drugs to reach their target blood pressure (BP), increasing the risk of side effects [1, 2]. Considering this limitation, there is evidence that the association of antihypertensive drugs with non-pharmacological interventions based on aerobic training can improve the clinical management of SAH [3]. In addition, exercise training promotes fewer adverse reactions, is economically viable, and can interact additively or synergistically with antihypertensive drugs [3].

Although aerobic training is effective to attenuate SAH, physiological mechanisms involved in this effect have not been elucidated. Vascular function adjustments are proposed as potential effects of aerobic exercise training, especially considering that endothelial dysfunction is a characteristic change in hypertensive subjects [4], who exhibit increased vasoconstrictor tone and peripheral vascular resistance [5]. There is evidence that aerobic exercise training can attenuate endothelial dysfunction and up-regulate endothelial nitric oxide synthase and superoxide dismutase expression and activity, as well as improving vascular antioxidant capacity, effects that by stimulating vasodilation may help to mitigate SAH [6]. In addition, down-regulation of vasoconstrictor endothelin-1 gene expression and reduced protein bioavailability, as well as reduced levels of angiotensin II type 1 receptor (required for angiotensin II-mediated vasoconstriction), were also found after aerobic exercise training [6].

Platelet-activating factor (PAF) is an endogenous phospholipid released from different biological sources, including platelets, polymor-phonuclear leukocytes, macrophages, endothelial cells, and vascular smooth muscle cells [7]. This factor is recognized for triggering a wide spectrum of biological activities, including pro-inflammatory and hypotensive effects [7]. A study found long-lasting PAF-induced hypotension, dose-dependent, in both normotensive and spontaneously hypertensive rats, guinea pigs, rabbits and dogs [8]. As PAF is a phospholipidic mediator synthesized by endothelial and smooth muscle cells, its hypotensive effect may be attributed to the dilation of resistance vessels, suggesting its involvement in modulating endothelial dysfunction and vascular smooth muscle tone [7]. Furthermore, Lopes-Martins et al. found that intravenous administration of the PAF receptor antagonist WEB 2086 significantly potentiated the increased systemic vascular resistance induced by noradrenaline or by central activation of the sympathetic nervous system with glutamate, demonstrating that PAF is involved in the modulation of vasomotor tone and, hence, arterial blood pressure by a negative feedback mechanism triggered by important increases in vascular tone [9]. In addition, fatty acids of the PAF family containing C16 (PAFC16) or C18 (PAFC18) carbon chains promoted renal vasodilation with a decrease in renal vascular resistance and systemic hypotension [10].

Thus, these early studies suggest that the hypotensive effect of PAF may be by the actions on the central or autonomic nervous systems, or mainly by direct actions on the peripheral blood vessels.

PAF is synthesized by two pathways: the “remodelling pathway” is mainly associated with PAF production by activated leukocytes. This pathway requires a tightly coupled reaction between phospholipase A2 and acetyl CoA: lkyl-sn-glycero-hosphorylcholine 2-O-acetyltransferase [7]. The second mechanism is mediated by the “*de novo* pathway”. This mechanism is primarily identified in the kidney and central nervous system and requires the synthesis of 1-O-alkyl- cetylglycerol, which is then converted to PAF by a specific dithiothreitol-insensitive CDP-choline: lkyl-cetyl-snglycerol choline phosphotransferase [7].

Some studies have shown that PAF from both pathways is involved in the control of several pathologies. Jeong et al. [11] demonstrated that PAF treatment induced a protective effect against mortality from endotoxic shock in mice. This effect was associated with the inhibition of apoptosis by lymphocytes and correlated with significantly decreased production of inflammatory mediators such as TNF-α, IL-1β, IL-12, and IFN-γ, and increased production of the anti-inflammatory cytokine IL-10. In addition, another study found that TNF-α promotes intestinal mucosal repair in parallel to upregulation of the PAF receptors in the intestinal epithelium [12]. Furthermore, PAF receptors are involved in enhanced apoptosis, via activation of the factor nuclear kappa B (NF-κB) pathways, in the same type of cancer [13, 14].

Studies have also demonstrated the importance of the cardioprotective effect of PAF, especially at low levels, both in reducing the extent of myocardial injury after infarction and in improving the recovery of left ventricular developed pressure and left ventricular end-diastolic pressure after an ischaemic episode [15].

Studies investigating the potential effects of exercise training on PAF activation are scarce. Although increased plasma levels of PAF after acute eccentric exercise have been identified in a previous study [16], the impact of chronic aerobic exercise on the relationship between PAF and BP remains overlooked. Thus, we used a randomized clinical trial framework to investigate this relationship, especially considering the potential implications of PAF in aerobic training-induced hypotensive effects in subjects with SAH.

## MATERIALS AND METHODS

### Study design and sample

This randomized controlled trial was previously registered in the Clinical Trials Register (ID: RBR-2vk9t4, registered in June 2019). Data were collected from August 2019 to March 2020. Furthermore, this study was approved by the local Ethics Research Committee for Human Research (protocol 039/2015, CAAE: 09381018.6.0000.5142), which was conducted by the Declaration of Helsinki and the CONSORT recommendations for non-pharmacological trials [17].

Hypertensive subjects referred to the local Cardiac Rehabilitation Centre were included in this study. Systemic arterial hypertension was diagnosed by a certified cardiologist according to well-established criteria from the European Society of Hypertension and the European Society of Cardiology [18]. Volunteers of both sexes, between 40 and 80 years old, able to perform aerobic physical activity and who did not perform regular physical activities in the previous three months, were included in the study. The following exclusion criteria were adopted: the presence of diseases that prevent or interfere with exercise practice; inflammatory or infectious disease; chronic pain; history of surgery or fractures in the lower limbs in the last six months; neurological diseases or sequelae, unstable angina, thrombophlebitis, recent thromboembolism, 3^rd^-degree atrioventricular block, uncontrolled cardiac arrhythmia, haemodynamic instability, and uncontrolled SAH.

All potentially eligible subjects were contacted. Those interested in participating were invited to attend the physical examination to assess the inclusion and exclusion criteria. All participants were informed about the study and provided informed consent. An evaluator blinded to treatment groups performed the physical examination. Demographic and anthropometric data, antihypertensive drug use, and date of SAH diagnosis were collected at baseline before randomization and after randomization by the same blinded evaluator.

Diastolic (DBP) and systolic (SBP) blood pressures and plasma PAF levels were the primary outcomes evaluated in this study. DBP and systolic SBP blood pressures were analysed in participants sitting at rest for 10 minutes. A calibrated aneroid sphygmomanometer coupled to an appropriately sized brachial blood pressure cuff was used. All measurements were collected on the left arm at the heart level. The average of two readings was recorded as the blood pressure value for each subject. Recommended procedures were used to select the position and sizes of sphygmomanometers [19].

The following secondary outcomes were adopted: functional capacity, human activity profile, quality of life, maximum inspiratory pressure (MIP), maximum expiratory pressure (MEP), and cytokine plasma levels. The six-minute walking test (6-MWT) was used to assess functional capacity, following the guidelines of the American Thoracic Society [20]. The test was conducted along a 3 eter indoor walkway marked every 5 m with non-slip coloured tape glued to the floor. Before and immediately after the test, the heart rate (HR), SBP, DBP, and perceived exertion scores were recorded. Using a wristwatch, resting HR was measured for 60 seconds by palpating the radial artery. This measurement was preceded by at least 5 minutes of sitting rest.

### Human activity profile

Each participant answered the Human Activity Profile (HAP) questionnaire at the beginning of the study [21]. This instrument includes 94 items related to activities such as self-care, transportation, home maintenance, entertainment/social, and physical exercises. Three responses are possible for each item: “still doing the activity”, “have stopped doing the activity”, and “never did the activity”. Then, an adjusted activity score (AAS) was calculated to determine the respondents’ activity levels, which were classified into impaired (AAS < 53), moderately active (AAS between 53–74), and active (AAS > 74).

### Quality of life

The SF-36 (Short Form Health Survey) questionnaire was used to assess the quality of life [22]. This tool comprises 36 items, which are mostly assigned to one of eight health domains covering aspects of physical and mental health, as follows: physical functioning (10 items), physical role functioning (4 items), bodily pain (2 items), general health perceptions (5 items), vitality (5 items), social role functioning (2 items), emotional role functioning (3 items), and mental health (5 items). Higher scores in each domain indicate better health status. Scores range from 0 to 100, so results closer to 0 are less favourable to health status and scores closer to 100 are more favourable. As the questionnaire was administered, each question was read and clarified by the evaluator. After the participants answered all the questions, the score was calculated using the SF36^+^ software.

### Respiratory muscle strength

Maximal inspiratory and expiratory pressures were evaluated as indicators of respiratory muscle strength. These parameters were evaluated with the individual in the sitting position using an analogue manovacuometer with a scale ranging from -120 to +120 cmH_2_O. A silicone mouthpiece adapter with a distal hole, which was sealed during each respiratory manoeuvre, was used to facilitate pressure recording [23].

### PAF biochemical assay

PAF was analysed according to the Bligh and Dyer method [24]. Briefly, a 4 mL plasma sample was obtained from the cubital vein and immediately placed in a Vacutainer tube (BD Biosciences, USA), transferred to another 2 mL tube containing methanol, and spiked with 10 *μ*L of internal standard solution (IS). Samples were then homogenized for 3 minutes at 30 Hz (Bead Ruptor 96, Omni Inc), followed by chloroform addition, and homogenization for more 3 minutes at 30 Hz. The homogenate was transferred to a glass tube with 250 *μ*L of chloroform and 500 *μ*L of ultrapure water. The samples were vortexed for 10 minutes and centrifuged at 3000 g for 10 minutes at 4°C. The lower phase (organic phase) was collected. The upper phase was re-extracted with 250 *μ*L of chloroform, and the lower phase was additionally collected. The lower phases were combined and dried in a vacuum centrifuge for 1 h at room temperature. Aliquots of this dried organic phase were re-suspended in 50 *μ*L MeOH/H_2_O (7:3, v/v) for liquid chromatography with tandem mass spectrometry (LC-MS/MS) analysis.

LC-MS/MS analysis was conducted using a High-Performance Liquid Chromatography (HPLC) system (Nexera X2, Shimadzu-Kyoto, HO, Japan) coupled to a TripleTOF 5600+ mass spectrometer (Sciex-Foster, CA, USA) equipped with a Turbo-V IonSpray and a Calibrant Delivery System (CDS). The APCI Positive Calibration Solution (SCIEX) was used. The Ascentis C18 column (15 cm × 2.1 mm, 2.7 *μ*m; Supelco) was used for chromatographic separation, according to the method described by Godzien et al. [25], with some modifications reported below. Briefly, elution was performed using 0.1% formic acid solution prepared in water (v/v) (phase A) and 0.1% formic acid prepared in methanol (v/v) (phase B), and specific gradient conditions as follows: 0 to 1.0 min, 30% B; 1.0 to 3.5 min, 80% B; 3.5 to 12.0 min, 100% B. The column temperature was maintained at 40°C, the flow rate at 0.5 mL/min, and the samples were maintained at 4°C in the auto-sampler. The parameters used in the mass spectrometer were: nebulizer gas (GS1), 50 psi; turbo-gas (GS2), 50 psi; curtain gas (CUR), 25 psi; electrospray voltage (ISVF), 4.5 kV; turbo ion spray source temperature, 500°C, dwell time, 100 ms, and mass resolution < 2 ppm. Data acquisition was performed using the Analyst software (Sciex-Foster, CA, USA). Data were processed using PeakView 2.1 and MultiQuant 3.0.2 software (Foster, CA, USA) for multiple fragment ions’ extraction, generation of MRMHR channels, and quantitative analysis. The MRMHR target parameters used were: PAF-C16:0 (524.3678/180.0731) and PAF-C18:1 (550.3859/184.0729) – parent ion and precursor ion (m/z), respectively.

### Cytokine immunoassay

In addition to its potential involvement in SAH pathophysiology, PAF is a lipid mediator with several inflammatory and non-inflammatory roles, so its production may occur through the “remodelling pathway” under inflammatory conditions. Thus, cytokine plasma levels were also quantified. For that, aliquots of the same plasma samples obtained to evaluate PAF were frozen at -80°C and later used to quantify circulating cytokines. Plasma was obtained after centrifuging the blood at 500 x*g* and 4°C in the absence of an anticoagulant. A Cytometric Bead Array (CBA) human cytokine kit (BD Biosciences, USA) was used to quantify IL-2, IL-4, IL-6, TNF-α, and IFN-ᵧ serum levels following the manufacturer’s instructions. The concentration (pg/mL) of each cytokine was calculated from a standard curve of recombinant molecules (CellQuest, BD Biosciences, USA) using CBA software. The FACSCalibur cytometer (BD Biosciences, USA) was used for all readings.

### Aerobic training protocol

Before starting the aerobic training, eligible participants were randomized to the intervention (IG) or control (CG) group. A research physiotherapist carried out the recruitment and evaluation of the participants, another carried out the randomization, and two other physiotherapists carried out the intervention. The allocation of participants was done using simple randomization by flipping a coin. IG participants performed three 5 inute sessions of treadmill aerobic exercise training (Movement RT250, USA) per week, on alternate days (Monday, Wednesday, and Friday), for 12 weeks in a total of 36 sessions. In this group, the training intensity was kept within 60–70% of the HR (target zone), which was calculated according to the Karvonen formula, with HRmax based on the formula 22 ge [26]. Heart rate was continuously measured using a precise heart rate sensor attached to a chest strap (Polar, model H10, NY, USA), from which data were transferred to a tablet via Bluetooth. Aerobic training was based on a inute warm-up, 4 inute target-zone training, and a final inute cool-down [27]. Training intensity was determined by a score ranging from 5 to 7 on the modified Borg’s perceived exertion scale for participants who did not achieve target HR [27]. Blood pressure, HR, and RPE were measured before, during (every 10 minutes), and after each exercise session. This exercise training was administered between 7:00 and 10:00 am. Participants in the CG group were instructed to perform only simple activities associated with daily living and were invited to participate in the cardiac rehabilitation programme after the study. All participants were reassessed twenty-four hours after the last training session or after 12 weeks for control volunteers. Blood samples for PAF and cytokine dosage were collected before the beginning of the aerobic training and 24 hours after the last session of the 12^th^ week.

### Statistical analysis

The sample size used was calculated considering the similarity in methodology and clinical outcomes evaluated in a previous study [28]. The sample size was calculated using the Gpower software (version 3.1.9.2, North Rhine-Westphalia, Germany), adopting a 5% significance level, 80% power, and a 0.8 correlation coefficient for all variables. The calculations were based on the mean SBP values obtained in the aforementioned study, which demonstrated the need for at least 76 participants, of which, when considering a possible average loss of 8%, according to the formula described by Whitley & Ball et al. [29], we arrive at a total of 82 participants, 41 in each group.

Patient characteristics were expressed as absolute and relative values. Data distribution was analysed using the Shapiro-Wilk test. Parametric data were expressed as mean and standard in addition to promoting a gain in physical conditioning, as found in the present study, in which the IG participants walked more metres in the 6MWT, while non-parametric data were presented as the median. Baseline and post-intervention data (clinical, cytokines, and quality of life) were compared using the paired t-test or Wilcoxon test for intragroup comparisons, and the independent t-test or Mann-Whitney test for intergroup comparisons. One-way ANOVA followed by the Bonferroni post hoc test was used to compare PAF levels. Variables potentially associated with PAFC16:0 and PAFC18:1 plasma levels were explored by multivariate linear regression. The “backward” method was used to adjust the regression model. The linear relationship between independent and dependent variables, absence of multicollinearity, presence of homoscedasticity, independent distribution of errors, and normality of residuals were assumptions observed and respected in the final regression model. All analyses were performed using SPSS Statistics for Windows (version 23.0, IBM, Armonk, USA). The significance level for all statistical tests was set at 5%, and a statistician blinded to the groups included in the study analysed all data. The effect size was calculated by using Cohen’s d, and the results were interpreted following Cohen as follows: small (0.21–0.49), medium (0.50–0.79), or large (≥ 0.80) [30].

## RESULTS

**FIG. 1:**
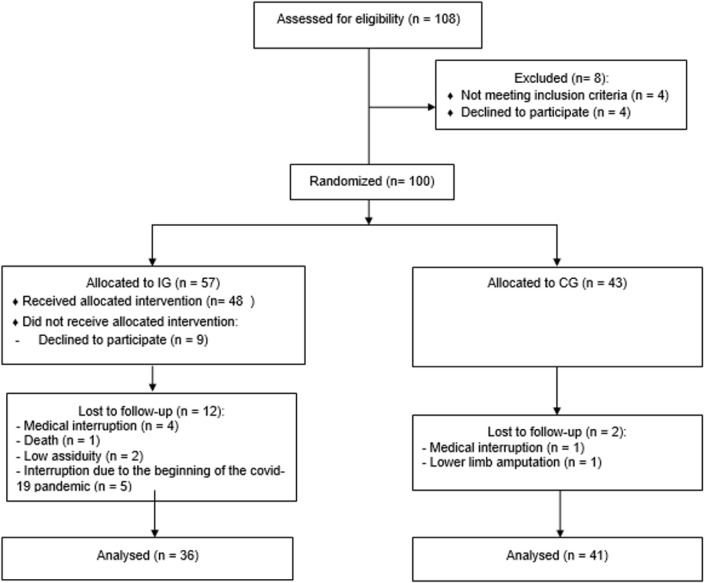
CONSORT diagram showing the participants’ progress through the trial.

The flowchart indicating patients’ progression through the study is shown in Figure 1. Fifty-seven participants were initially allocated to the intervention group (IG) and forty-three to the untrained control group (CG). Thirty-six trained and forty-one untrained participants completed the study and were evaluated at the end of treatment.

The investigated sample was mainly composed of women aged 66.51 ± 7.53 years. The average of participants classifies them as overweight (BMI = 29.50 ± 4.51 kg/m^2^) (Table 1). Regarding alcohol and tobacco consumption, 23.4% of the sample were alcohol consumers and 20.8 were ex-alcohol consumers; 18.2% were smokers and 53.2% were ex-smokers. Both alcohol consumers and exalcohol consumers were classified as low-consumption, evaluated by the AUDIT (Alcohol Use Disorders Identification Test) [31]. Angiotensin-receptor blockers (ARB) (68.83%) and diuretics (45.45%) were the main drugs used. Diabetes mellitus was the most frequent comorbidity (61.03%). Most participants (67.5%) were classified as moderately active according to the physical activity profile assessed by the HPA questionnaire (Table 1).

**TABLE 1:** Baseline characteristics of the participants.

Variables	IG (n = 36)	CG (n = 41)	Total (n = 77)
Age (years)	66.97 ± 6.99	66.12 ± 8.03	66.51 ± 7.53
Men, n (%)	15 (41.7%)	16 (39.0%)	31 (40.3%)
Women, n (%)	21 (58.3%)	25 (61.0%)	46 (59.7%)
BM (Kg)	76.15 ± 13.89	76.18 ± 14.62	76.17 ± 14.19
BMI (Kg/m^2^)	29.65 ± 3.47	29.37 ± 5.30	29.50 ± 4.51
AC (cm)	100.63 ± 1.71	98.78 ± 1.82	99.70 ± 1.52
SBP (mmHg)	133.05 ± 2.38	133.41 ± 2.48	133.23 ± 2.41
DBP (mmHg)	82.22 ± 1.44	81.70 ± 1.43	81.96 ± 1.39
HAP scoring	59.11 ± 3.22	59.39 ± 2.49	59.26 ± 1.99
Life habits, n (%)			
Alcohol consumers	8 (22.2)	10 (24.4)	18 (23.4)
Ex-alcohol consumers	11 (30.6)	5 (12.2)	16 (20.8)
Non-alcohol consumers	17 (47.2)	26 (63.4)	43 (55.8)
Smoker	6 (16.7)	8 (19.5)	14 (18.2)
Ex-Smoker	19 (52.8)	22 (53.7)	41 (53.2)
Non-Smoker	11 (30.6)	11 (26.8)	22 (28.6)
Medications, n (%)			
ARBs	22 (61.11)	31 (75.60)	53 (68.83)
β-Blockers	15 (41.66)	16 (39.02)	31 (40.25)
Diuretics	21 (58.33)	14 (34.14)	35 (45.45)
ACEi	7 (19.44)	7 (17.07)	14 (18.18)
CCBs	8 (22.22)	14 (34.14)	22 (28.57)
Associated pathologies, n (%)			
Diabetes Mellitus	21 (58.33%)	26 (63.41%)	47 (61.03%)
Dyslipidemia	5 (13.88%)	9 (21.95%)	14 (18.18%)
CABG	2 (5.55%)	0	2 (2.59%)
MI	8 (22.22%)	1 (2.43%)	9 (11.68%)
Hypothyroidism	6 (16.66%)	8 (19.51%)	14 (18.18%)
Stable angina	1 (2.77%)	0	1 (1.29%)

BM = body mass; BMI = body mass index; AC = abdominal circumference; SBP = systolic blood pressure; DBP = diatolic blood presure; HAP = human activity profile; ARBs = angiotensin-II receptor blockers; ACEi = angiotensin-converting enzyme; CCBs = calcium-channel blockers; CABG = coronary artery bypass graft surgery; MI = miocardial infaction. Data are presented as frequency or mean ± SEM.

**FIG. 2:**
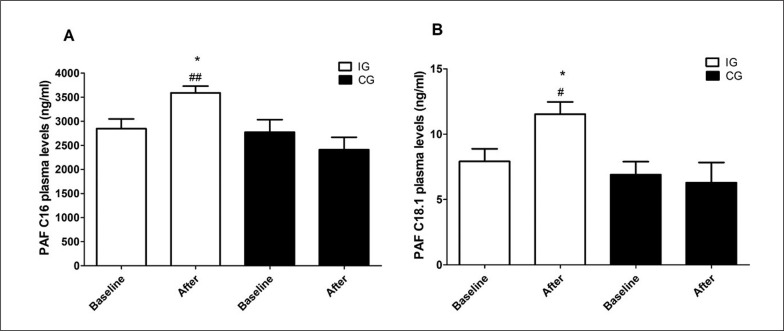
Effect of aerobic training on plasma levels of PAFC16 and PAFC18.1. Data are presented as mean+SEM. *p < 0.05 indicates statistically significant differences compared to baseline. ^#^p < 0.05 and ^##^p < 0.01 indicate statistically significant differences between the intervention group (IG) and the control group (CG). One-way ANOVA followed by Bonferroni post-test.

**FIG. 3:**
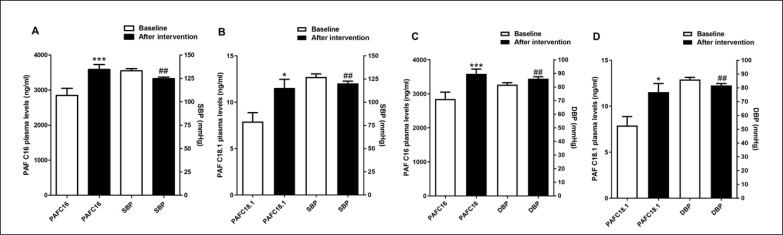
Reduction of blood pressure levels and increase of PAF levels after aerobic training. Data are presented as mean+SEM. *p < 0.05 and ***p < 0.01 indicate statistically significant differences compared to baseline. ^##^p < 0.01 indicates statistically significant differences between the intervention group (IG) and the control group (CG). One-way ANOVA followed by Bonferroni post-test.

As indicated in Table 2, post-intervention arterial blood pressure levels were significantly reduced [124.72 ± 1.66 vs. 133.05 ± 2.38 (95% CI 2.93–13.72, p = 0.048) for SBP and 76.94 ± 1.48 vs. 82.22 ± 1.44 (95% CI 4.82–5.73, p = 0.034) for DBP] in trained subjects compared to baseline. The effect size, as measured by Cohen’s d, was d = 4.12 and 3.60, respectively, indicating a large effect. Intergroup comparison indicated that trained participants exhibited reduced delta levels after 12 weeks [-8.33 ± 2.65 vs. 1.43 ± 2.47 (95% CI -17.00 – -2.53, p = 0.049) for SBP and -5.27 ± 2.3 vs. 4.36 ± 1.56 (95% CI 4.01–4.70, p = 0.02) for DBP] (Table 2). The effect size for intergroup comparisons of blood pressure values also presented a large effect (d = 5.18 for SBP and d = 6.21 for DBP). Thus, this result suggests that the aerobic training protocol was effective in reducing blood pressure in hypertensive subjects. Furthermore, PAF plasma levels were significantly increased in trained subjects compared to baseline [11.51 ± 4.51 vs. 7.91 ± 4.6 (95% CI -7.15 – -0.07, p < 0.004, d = 0.90 for PAFC18:1 and 3587.09 ± 677.69 vs. 2848.61 ± 957.63 (95% CI-139.4 – -82.49, p < 0.004, d = 0.89 for PAFC16:0)] and to untrained participants [11.51 ± 4.51 vs. 6.28 ± 4.6 (95% CI (95% CI 0.52 to 9.95, p < 0.05, d = 0.74 for PAFC18:1 and 3587.09 ± 677.69 vs. 2410.5 ± 770.83 (95% CI 301.9 to 2051, p < 0.001, d = 1.68 for PAFC16:0)] after 12 weeks (Figure 2). According to Cohen’s d, we considered the effect size of the results found with plasma PAF levels to be large, except for the comparison of PAFC18 levels between groups after 12 weeks, which was medium. Decreased blood pressure values and increased PAF levels after the intervention are also shown in Figure 3. The distance walked in the 6MWT [406.26 ± 17.95 vs. 491.82 ± 18.71 (95% CI-118.70 – -52.41, p < 0.05, d = 4.70)] and MEP [92.61 ± 4.57 vs. 107.69 ± 2.78 (95% CI -23.95 – -6.21; d = 4.10) were also increased comparing the post-training results with baseline and with the delta values registered in the untrained group [85.56 ± 16.32 vs. -7.0 ± 38.35 (95% CI 59.65–126.66, p < 0.04, d = 5.27) for 6MWT and 15.08 ± 4.36 vs. 0.26 ± 2.25 (95% CI 4.50–25.07, p < 0.01, d = 0.99) for MEP] (Table 2). These previous results with MEP and 6MWT show a large effect size. The intervention proved to be effective in increasing circulating PAF levels in addition to controlling blood pressure, exerting a beneficial effect by improving functional capacity and expiratory muscle strength in trained participants. Conversely, cytokine levels were similar in both groups (Table 2).

**TABLE 2:** Clinical variables, quality of life and cytokines levels of participants at baseline and post 12 weeks.

	IG (n = 36) Baseline Post Sig			CG (n = 41) Baseline Post Sig Clinical variables			IG vs CG IG delta CG delta Sig		
**Clinical variables**									
BM (Kg)	76.15 ± 2.31	76.05 ± 2.32	-1.01-1.21^a^	76.18 ± 2.28	75.75 ± 2.40	-0.58-1.46^a^	-0.10 ± 0.55	-0.43 ± 0.50	-1.15-1.82^c^
BMI (Kg/m^2^)	29.65 ± 0.57	29.37 ± 0.62	-0.40-0.94^a^	29.37 ± 0.82	29.29 ± 0.89	-0.31-0.46^a^	-0.27 ± 0.33	-0.07 ± 0.19	-0.94-0.54^c^
AC (cm)	100.63 ± 1.71	100.06 ± 2.24	-2.42-3.56^a^	98.78 ± 1.82	99.80 ± 1.95	-0.28-2.33^a^	-0.56 ± 1.47	-1.02 ± 0.64	-2.62-3.53^c^
SBP (mmHg)	133.05 ± 2.38	124.72 ± 1.66	2.93-13.72^a,*^	133.41 ± 2.48	134.85 ± 2.56	-6.44-3.57^a^	-8.33 ± 2.65	1.43 ± 2.47	-17.00 – -2.53c,^*^
DBP (mmHg)	82.22 ± 1.44	76.94 ± 1.48	4.82-5.73^b,**^	81.70 ± 1.43	86.07 ± 1.47	-6.24 – -4.29b,^**^	-5.27 ± 2.3	4.36 ± 1.56	4.01-4.70d,^**^
HR (bpm)	70.86 ± 1.97	68.83 ± 1.90	-1.48-5.53^a^	72.24 ± 1.74	71.95 ± 2.07	-2.97-3.56^a^	-2.20 ± 1.72	-0.29 ± 1.61	-6.45-2.98^c^
MIP (cmH_2_O)	-91.33 ± 4.74	-99.11 ± 3.88	-16.86-1.31^a^	-94.46 ± 4.21	-91.24 ± 4.28	6.40-9.13^b^	7.77 ± 4.47	-3.22 ± 3.61	-4.02 – -2.41d
MEP (cmH_2_O)	92.61 ± 4.57	107.69 ± 2.78	-23.95 – -6.21^a,*^	94.31 ± 2.96	94.61 ± 3.54	-6.26-5.67^a^	15.08 ± 4.36	0.29 ± 2.95	4.50-25.07c,^*^
6-MWT (m)	406.26 ± 17.95	491.82 ± 18.71	-118.70 – -52.41^a,*^	424.02 ± 9.86	416.92 ± 9.69	-5.00-19.20^a^	85.56 ± 16.32	-7.0 ± 38.35	59.65-126.66^c,*^

**Quality of life (score 0 to 100)**									
FC	67.22 ± 3.63	75.02 ± 3.98	-15.77–0.^a^	75.97 ± 3.23	76.58 ± 3.56	-7.38–6.16^a^	7.80 ± 3.92	0.61 ± 3.35	-0.13–1.35^d^
LPA	50.02 ± 6.05	72.19 ± 5.53	3.16–6.43^b,**^	56.78 ± 5.73	63.29 ± 6.0	-19.94–24.37^b^	22.16 ± 7.24	6.51 ± 6.69	-3.97–35.28^c^
Pain	54.69 ± 3.95	68.38 ± 4.36	-24.27 – -3.11^a,*^	54.29 ± 4.33	57.58 ± 4.09	-10.49–3.91^a^	13.69 ± 5.21	3.29 ± 3.56	-1.92–22.73^c^
GH	66.02 ± 3.32	76.91 ± 2.94	-18.32 – -3.45^a,*^	63.22 ± 2.82	56.51 ± 3.32	0.59–12.82^a,*^	10.88 ± 3.66	-6.70 ± 3.02	8.20–26.98^c,*^
VIT	59.75 ± 3.89	75.13 ± 2.74	-23.09 – -7.68^a,*^	59.63 ± 3.92	59.92 ± 3.81	-6.09–5.50^a^	15.38 ± 3.79	0.29 ± 2.86	5.75–24.44^c,*^
AS	67.69 ± 5.09	83.02 ± 3.77	-25.00 – -5.65^a,*^	77.58 ± 3.28	80.90 ± 4.24	-13.87–16.78^b^	15.33 ± 4.76	3.31 ± 4.39	-0.88–24.91^c^
LAE	48.88 ± 7.18	65.72 ± 6.30	-14.87–18.80^b^	40.65 ± 6.21	60.48 ± 6.75	14.10–19.55^b,**^	16.83 ± 8.34	19.82 ± 6.85	-18.28–21.35^d^
MH	68.19 ± 3.87	77.94 ± 3.18	-16.30 – -3.19^a,*^	68.12 ± 3.43	68.58 ± 3.08	-5.19–4.26^a^	9.75 ± 3.23	0.46 ± 2.34	1.47–17.10^c,*^

**Cytokines levels**									
INF-y (pg/mL)	0.00 (2.48)	0.00 (2.63)	-0.85–0.85^b^	0.00 (0.00)	0.00 (0.00)	-0.00–0.00^b^	-0.00–0.0	0.00 (0.00)	-0.00–0.00^d^
TNF-α (pg/mL)	0.16 (0.31)	0.16 (0.36)	-0.09–0.09^b^	0.00 (0.16)	0.16 (0.20)	-0.02–0.02^b^	0.00 (0.08)	0.16 (0.20)	-0.11–0.20^d^
IL-10 (pg/mL)	0.23 (0.49)	0.10 (0.32)	-0.05–0.21^b^	0.10 (0.23)	0.10 (0.23)	-0.05–0.05^b^	0.00 (-0.16)	0.00 (-0.13)	-0.02–0.02^d^
IL-6 (pg/mL)	1.66 (2.01)	1.53 (1.19)	-0.49–0.75^b^	1.53 (1.29)	0.81 (1.59)	-0.05–0.21^b^	0.08 (-0.41)	0.00 (-0.27)	-0.06–0.06^d^
IL-4 (pg/mL)	0.12 (0.62)	0.06 (0.62)	-0.10–0.22^b^	0.12 (0.12)	0.00 (0.12)	-0.04–0.04^b^	0.00 (-0.14)	0.00 (-0.12)	-0.02–0.02^d^
IL-2 (pg/mL)	0.00 (0.32)	0.32 (0.32)	-0.40–0.23^b^	0.00 (0.32)	0.32 (0.32)	-0.10–0.10^b^	0.00 (0.32)	0.00 (0.32)	-0.07–0.07^d^

BM = body mass; BMI = body mass index; AC = abdominal circumference; SBP = systolic blood pressure; DBP = diatolic blood pressure; MIP, maximal inspiratory pressure; MEP = maximal expiratory pressure; 6-MWT = six-minute walking test; FC = functional capacity; LPA = Limitation of physical aspects; GH = general health; VIT = vitality; AS = social aspects; LEA = limitation of emotional aspects; MH = mental health; INF-ᵧ = Interferon-gamma; TNF-α = Tumor Necrosis Factor-alpha; IL-10 = Interleukin 10; IL-6 = Interleukin 6; IL-4 = Interleukin 4; IL-2 = Interleukin 2. Data indicated as the mean and standard error of the mean, or median and interquartile range.

a Paired T test (IC95%);

b Wilcoxon test (valor p);

c Independent T test (IC95%);

d Mann–Whitney test (p value).

* and

** indicate statistical difference for IC95%.

Multiple linear regression analysis indicated that MEP was the strongest predictor (F [3.19] = 6.322; p = 0.001; R^2^ adjusted = 0.499) for PAFC16:0 plasma levels in subjects undergoing aerobic training. SBP (p = 0.436) and DBP (p = 0.085) showed no significant association with PAFC16:0. SBP (p = 0.742), DBP (p = 0.269) and MEP (p = 0.935) also did not show a significant association with PAFC18.1. It is important to highlight that the number of plasma samples collected for both cytokine and PAF measurements was reduced (29 samples in the control group and 27 in the intervention group). 12 participants refused to collect blood in the CG and 9 in the IG.

The trained subjects also showed a significant improvement in quality of life when the domains limitation of physical aspects [72.19 ± 5.53 vs. 50.02 ± 6.05 (95% CI 3.16–6.43, p = 0.006, d = 2.50)], pain [68.38 ± 4.36 vs. 54.69 ± 3.95 (95% CI -24.27–-3.11, p = 0.03, d = 3.29)], general health [76.91 ± 2.94 vs. 66.02 ± 3.32 (95% CI -18.32 – -3.45, p = 0.001, d = 3.47)], vitality [75.13 ± 2.74 vs. 59.75 ± 3.89 (95% CI -23.09 – -7.68, p = 0.002, d = 4.64)], social aspects [83.02 ± 3.77 vs. 67.69 ± 5.09 (95% CI -25.00 – -5.65, p = 0.004, d = 3.45)] and mental health [77.94 ± 3.18 vs. 68.19 ± 3.87 (95% CI -16.30 – -3.19, p = 0.02, d = 1.78)] were compared to baseline (Table 2). In addition, untrained subjects presented a worsening in the domain general health status [63.22 ± 2.82 vs. 56.51 ± 3.32 (95% CI 0.59–12.82, p = 0.03, d = 2.18)] and a better score in the domain limitation of emotional aspects [60.48 ± 6.75 vs. 40.65 ± 6.21 (95% CI 3.16–6.43, p = 0.01, d = 3.06)] compared to baseline. From the intergroup comparison, trained subjects obtained better quality of life delta scores in the domains of general health [10.88 ± 3.66 vs. -6.70 ± 3.02 (95% CI 8.20–26.98, p = 0.005, d = 3.25)], vitality [15.38 ± 3.79 vs. 0.29 ± 2.86 (95% CI 5.75–24.44, p = 0.002, d = 4.68)], and mental health [9.75 ± 3.23 vs. 0.46 ± 2.34 (95% CI 1.47–17.10, p = 0.004, d = 2.99)] (Table 2). A large effect size was found in the previously presented results of the quality of life domains.

## DISCUSSION

The present study demonstrated that aerobic training was efficient in reducing blood pressure while increasing circulating PAF levels in hypertensive individuals. In addition, increased functional capacity, expiratory muscle strength, and quality of life were verified by subjects undergoing aerobic training.

The study was composed mainly of overweight elderly subjects, most of them women. Corroborating the current evidence, SAH is more prevalent in the elderly population [32], and this disease is closely correlated to vascular stiffness caused by calcium accumulation, smooth-muscle hyperplasia within the tunica media, as well as quantitative and qualitative changes in vascular collagen [33]. In addition, overweight is directly related to SAH development [34]. In general, SAH rates are higher in women than men over 65 years old, and the hormonal imbalance associated with menopause seems to be related to this higher prevalence [35]. Smoking and alcohol intake are also directly related to SAH development [36]; these were frequent habits among the participants in this study. Although the HAP questionnaire indicated that most study participants were moderately active, the data previously presented showed that they had several risk factors for the development of SAH and other cardiovascular diseases.

Interestingly, the aerobic training protocol developed in this study proved to be a relevant treatment strategy, exerting a marked impact on the physical, functional, and psychological aspects evaluated. In addition to controlling blood pressure, aerobic training improved physical performance, expiratory muscle strength, and quality of life in hypertensive participants. There is consistent evidence of exercise-induced blood pressure control [37]. However, the mechanisms involved in this effect are not completely understood. Thus, the involvement of PAF in mechanisms linked to SAH pathophysiology suggests a potential effect of this molecule on aerobic training-associated blood pressure control [38, 39]. Accordingly, there is evidence that PAF exerts a hypotensive effect [7, 9, 10].

During exercise, there is an increase in the activation of the sympathetic autonomic nervous system, mainly to maintain cardiac output under the demand imposed by the intensity [40]. Furthermore, previous evidence has shown that PAF is released in response to sympathetic activity, primarily as negative feedback to initial vasoconstriction [9]. Thus, by a probable mechanism of increasing the energy supply to the tissues under the demand for exercise, the PAF induces a decrease in peripheral vascular resistance. Few mechanisms have been elucidated with PAF-induced hypotension. In addition, studies have ruled out the involvement of the renin-angiotensin system, muscarinic, β-adrenergic, dopaminergic, eicosanoids, calcium influx, thyrotropin-releasing hormone, steroids, or histaminergic mechanisms [7]. Thus, the mechanisms demonstrated in the hypotensive effect of this substance have been by modulation of vascular smooth muscle tone via negative feedback mechanism triggered by central activation of the sympathetic nervous system or peripherally promoting renal vasodilation [7, 9, 10], which may justify their release during the exercise.

In resting conditions, experimental studies have shown the hypotensive effect of PAF. A study found a dose-dependent decrease in mean arterial blood pressure in a model of spontaneously hypertensive rats after intravenous PAF infusion [38]. A similar effect was demonstrated in dogs by another study in the same year [39]. In addition, administration of the PAF receptor antagonist CV-3988 increased blood pressure in rats [41]. These findings suggest that PAF is involved in blood pressure control under normal conditions and during SAH.

We found a significant increase in PAFC16:0 and PAFC18:1 plasma levels in trained subjects. Thus, considering the increased plasma PAF levels after aerobic exercise, to the best of our knowledge this was the first clinical study to investigate and find an increase in this substance after aerobic training in humans. Haynes et al. [42] documented increased post-exercise PAF levels. However, they used eccentric exercise and evaluated this effect on platelet-mediated thrombotic risk. Platelet-activating factor (PAF) is a family of lkyl-cetyl glycerolhosphorylcholines that has a fatty acid linked by an ether bond at the sn-1 position (fatty acids containing C16 or C18 carbon chains are the most abundant), an acetyl group at the sn-2 position, and a polar head of phosphorylcholine at the sn-3 position [43]. The modulating effect of PAF on blood pressure was also demonstrated by intra-arterial infusion of PAFC16:0 and PAFC18:1, which was able to induce renal vasodilatation and systemic hypotension in rats [42]. PAF is synthesized by two markedly different pathways known as the “*de novo*” and “remodelling” pathways. The remodelling pathway is responsible for the proinflammatory PAF production in acute and chronic inflammations [44]. Conversely, the *de novo* pathway was initially thought to be responsible for constitutive PAF production, maintaining basal PAF levels [45]. To produce its effects, PAF binds to specific receptors on smooth muscle cells, cardiomyocytes, neutrophils, monocytes-macrophages, eosinophils, Kupffer cells, and endothelial cells [7].

Although PAF is related to the continuous activation of inflammatory cascades during the development of inflammation-related chronic disorders [44], changes in anti-(IL-4, IL-10) and proinflammatory (IFN-γ, TNF-α, IL-2, IL-6) cytokine levels were not identified in the present study. Supporting our results, there is evidence that PAF can be produced in the non-inflammatory remodelling pathway via lysophosphatidylcholine acyltransferase 1 (LPCAT1), a member of the lysophospholipid acyltransferase family that is involved in PAF biosynthesis, which is neither activated nor up-regulated by inflammatory stimuli [45]. Furthermore, a study demonstrated that the PAF-induced hypotensive effect was prevented by Nω-nitro-L-arginine, an inhibitor of nitric oxide (NO) biosynthesis in endothelial cells, suggesting that NO, the “endothelium-derived relaxing factor”, may be involved in PAF-mediated SAH control [46]. In addition, evidence has shown the contribution of NO to the regulation of blood pressure after exercise training [47]. NO was found to be an endothelial-derived relaxing factor and may play an important role not only in endothelial relaxation but also in the prevention of endothelial cell dys-function [47]. Exercise-induced shear stress stimulates NO release, upregulating NO synthesis [48]. Thus, in addition to the non-inflammatory pathway, this previous evidence also indicates that PAF may also reduce blood pressure via NO; however, more studies are needed to evaluate this process after aerobic training, since an association between increased levels of PAF and reduced arterial blood pressure was not found in the present study.

Multiple regression analysis did not find an association between plasma PAF levels and post-intervention arterial blood pressure. Linear regression assumes that relationships between variables are linear and that errors are normally distributed and have constant variance. If these assumptions are not met, the regression results may be inaccurate [48]. Thus, the difference between the number of PAF samples and blood pressure may have influenced the linear regression results. The reduced PAF sample may also have been a limitation of the present study.

Furthermore, the increase in PAF levels after aerobic training may not be involved in the control of SAH, but with other beneficial effects that both exercise and PAF participate in, such as activation of the immune system, neuronal plasticity, or body weight control [49–52]; however, further studies are needed to evaluate this process.

A moderate determination coefficient (R^2^ = 0.499) was found between increased plasma PAF C16:0 levels and expiratory muscle strength. A study conducted by our research group also demonstrated increased expiratory muscle strength and reduced SBP levels in hypertensive subjects after eek aerobic exercise training [53]. In addition, another study using aerobic exercises for upper and lower limbs through virtual cardiac rehabilitation for 15 weeks found increased expiratory muscle strength and reduced DBP in hypertensive patients [23].

Despite the gain in expiratory muscle strength having been associated with the reduction in SAH, as demonstrated in previous studies, and with the increase in PAF levels in the present study, there are few data in the literature that support this effect, especially after exercise. A study found a significant increase of PAF in the bronchoalveolar lavage fluid of race-trained horses [54]. In addition, PAF is responsible for potentiating the phagocytosis activity exerted by alveolar macrophages and neutrophils, which is crucial for lung clearance [55, 56]. Although further studies are needed, based on the previous evidence, we can suggest that PAF contributes to lung ventilation, and consequently to respiratory muscle strength. Furthermore, as during exercise there is an increase in the release of this substance, its contribution to alveolar clearance can facilitate gas exchange and lung efficiency, improving the ventilatory machinery and consequently respiratory muscle strength.

### Limitations and future directions

Although the study demonstrated several benefits of aerobic training, some limitations must be considered. Accordingly, a group of normotensive subjects would be relevant to compare pre-and post-exercise PAF levels with the results recorded in hypertensive subjects. However, it was difficult to obtain the number of hypertensive participants according to the calculated sample size, so it would be even more difficult to include normotensive individuals. Although our sample complied with the sample size calculation, the number of participants was too small to expand the use of independent variables in the multiple regression model. Furthermore, several factors may have limited the observed association of increased PAF levels with the reduction in blood pressure, such as I) the intensity of the exercise, which was of low intensity; II) the duration of the aerobic training programme, which can be considered short, and the discrepancy between the BMI of the participants, since some were overweight and others normal weight, and III) a reduced sample for PAF dosage.

Future research, excluding these previous biases, will be necessary to evaluate the involvement of PAF in the control of hypertension induced by exercise, in which resistance exercise can also be an alternative.

## CONCLUSIONS

Taken together, our results indicated that the aerobic training protocol developed in this study had a beneficial impact on blood pressure, physical performance, respiratory muscle strength, and quality of life in hypertensive patients. In addition, increased circulating PAF levels in trained subjects were associated with increased expiratory muscle strength, requiring more studies in future to assess the participation of this substance in blood pressure control.
